# Effect of plasma exchange in acute respiratory failure due to Anti-neutrophil cytoplasmic antibody-associated vasculitis

**DOI:** 10.1186/s13054-018-2264-x

**Published:** 2018-12-04

**Authors:** Guillaume Geri, Benjamin Terrier, Farhad Heshmati, Hanafi Moussaoui, Julien Massot, Jean-Paul Mira, Luc Mouthon, Frédéric Pène

**Affiliations:** 1 Medical Intensive Care Unit, Cochin Hospital, Assistance Publique-Hôpitaux de Paris, Paris, France; 2 Paris Descartes University, Paris, France; 3 Department of Internal Medicine, Cochin Hospital, Assistance Publique-Hôpitaux de Paris, Paris, France

## Abstract

**Background:**

Acute respiratory failure related to diffuse alveolar hemorrhage (DAH) is a typical presentation of small-vessel vasculitis that requires prompt multidisciplinary management. The primary treatment is based on immunosuppressive drugs, whereas urgent plasma exchange has been proposed in case of life-threatening complications. We addressed the course of respiratory failure in 12 patients with ANCA-associated vasculitis-related DAH.

**Patients and methods:**

Observational retrospective case series performed in the medical ICU of a tertiary hospital in Paris, France. Consecutive patients with ANCA-associated DAH admitted to our ICU for acute respiratory failure and treated by plasmapheresis were included in the analysis. We evaluated the SpO_2_/FiO_2_ ratio and assessed the mechanical ventilation mode hourly for 7 days.

**Results:**

Twelve patients were included. Five of them required invasive mechanical ventilation. All patients were treated by plasma exchange in addition to a combination of glucocorticoids and immunosuppressive agents. Oxygenation improved over the first 7 days following initiation of plasma exchange, as shown by a dramatic increase in the median SpO_2_/FiO_2_ ratio from 183 [interquartile 137–321] to 353 [239–432] (*p* = 0.003), along with a decrease in the level of ventilatory support. All but one patient survived.

**Conclusions:**

A multimodal induction regimen combining immunosuppressants and plasma exchange may rapidly reverse the respiratory dysfunction in ANCA-associated vasculitis-related DAH.

Besides infectious complications, severe acute flares of ANCA-associated vasculitis are common reasons that warrant ICU admission [1]. Plasma exchange (PLEX) has been proposed as an urgent adjuvant treatment in patients with life-threatening organ dysfunctions [2, 3]. In order to explore this question, we conducted a retrospective monocenter study in our tertiary ICU. We included patients admitted to the ICU for acute respiratory failure related to DAH, diagnosed as ANCA-associated vasculitis, and who received urgent initiation of PLEX. DAH was defined by bilateral infiltrates on chest X-ray and macroscopically bloody bronchoalveolar lavage with hemorrhagic and siderophagic alveoliitis. PLEX was performed daily with 1.2 plasma volume plasmapheresis primarily substituted with fresh frozen plasma and then albumin 5% and fresh frozen plasma when needed to maintain a prothrombin time > 50% and a fibrinogen level > 1.5 g/L. The main outcome was the evolution of oxygenation over the first seven days, using the SpO_2_/FiO_2_ ratio. We present data as median [interquartile range] or number (percentage) as appropriate. *P* for trend for continuous variables was calculated using a Cuzick test.

Between 2006 and 2014, 12 patients were treated by PLEX in the ICU for ANCA-vasculitis with respiratory symptoms (Table 1). All patients received high-dose corticosteroids (≥ 1 mg/kg prednisone-equivalent) and additional immunosuppressive drugs, either cyclophosphamide (nine within 24 h before or after ICU admission and one after ICU discharge) or rituximab administrated in the ICU (*n* = 2). One patient died from refractory multiple organ failure related to septic shock. Invasive mechanical ventilation was required in five patients (two received high-frequency oscillation ventilation). One patient received adjuvant nitric oxide. Duration of invasive mechanical ventilation ranged from 6 to 20 days. Three patients successfully received non-invasive ventilation. Oxygenation improved over the first week, as shown by the increase in the SpO_2_/FiO_2_ ratio from 183 [137–321] to 353 [239–432] (*p* value for trend 0.003), along with a decrease in the level of ventilatory support (Fig. 1). In contrast, only one out of five patients could be weaned off dialysis.

**Table 1:** Characteristics of patients

Variable	All patients
	*n* = 12
Female gender	7 (58)
Age (years)	62.1 [49.3–71.6]
Small-vessel vasculitis	
Granulomatosis with polyangiitis	9 (75)
Microscopic polyangiitis	3 (25)
Vasculitis flare as first manifestation of the disease	11 (92)
Previous maintenance treatments	
Corticosteroids	2 (16)
Immunosuppressants	6 (50)
Organ involvement at ICU admission	
Pneumo-renal syndrome	9 (75)
Respiratory SOFA component	3 [2–4]
PaO_2_/FiO_2_ ratio	154 [61–386]
PaCO_2_ (mmHg)	35 [31–46]
Acute kidney injury requiring RRT	5 (42)
Renal SOFA component	2 [1–4]
Blood creatinine level (μmol/L)	222 [94–450]
Roteinuria (g/24 h)	1.5 [1.5–3]
Hemoglobin level (g/dL)	10.1 [8.2–10.7]
Therapeutics received in the ICU	
Number of plasmapheresis courses	6 [4–7]
Corticosteroids	12 (100)
Immunosuppressants	12 (100)
Invasive mechanical ventilation	5 (42)
ICU length of stay (days)^a^	11 [7–15]
In-ICU mortality	1 (8)

Continuous and categorical variables are described as median [interquartile range] and number (percentage), respectively

^a^In-ICU length of stay in patients with and without mechanical invasive ventilation were 15 [9–17] and 7 [4–12] days, respectively (*p* = 0.03)

*RRT* renal replacement therapy, *SOFA* sequential organ failure assessment

**Fig. 1:**
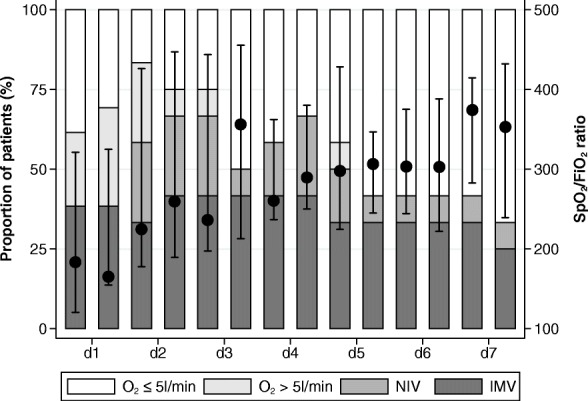
Evolution of respiratory dysfunction as assessed every 12 h over the first 7 days (*d1* to *d7*) from initiation of plasma exchange. *Black dots* and *lines* represent the SpO_2_/FiO_2_ ratio (median and interquartile range). Background histograms show the distribution of ventilatory support. *NIV* non-invasive ventilation, *IMV* invasive mechanical ventilation

In conclusion, this suggests the addition of PLEX results in fast respiratory recovery in most patients. This contrasts with the limited impact on renal function. The effects of PLEX are presumably related to fast removal of auto-antibodies as well as pro-inflammatory mediators likely to induce and/or sustain the increased permeability of the alveolo-capillar barrier.
